# The cost of uncomplicated childhood fevers to Kenyan households: implications for reaching international access targets

**DOI:** 10.1186/1471-2458-6-314

**Published:** 2006-12-29

**Authors:** Bruce A Larson, Abdinasir A Amin, Abdisalan M Noor, Dejan Zurovac, Robert W Snow

**Affiliations:** 1 Center for International Health and Development, Boston University, 85 East Concord Street, 5th Floor, Boston, MA 02118, USA; 2 Centre for Clinical Research, Kenya Medical Research Institute, PO Box 20778, Nairobi 00202, Kenya; 3 Department of Agricultural and Resource Economics, Unit 4021, University of Connecticut, Storrs, CT 06269, USA; 4 Malaria Public Health & Epidemiology Group, Centre for Geographic Medicine Research-Coast, Kenya Medical Research Institute/Wellcome Trust Research Programme, P.O. Box 43640, 00100 GPO, Nairobi, Kenya; 5 Centre for Tropical Medicine, University of Oxford, John Radcliffe Hospital, Headington, Oxford, OX3 9DU, UK

## Abstract

**Background:**

Fever is the clinical hallmark of malaria disease. The Roll Back Malaria (RBM) movement promotes prompt, effective treatment of childhood fevers as a key component to achieving its optimistic mortality reduction goals by 2010. A neglected concern is how communities will access these new medicines promptly and the costs to poor households when they are located in rural areas distant to health services.

**Methods:**

We assemble data developed between 2001 and 2002 in Kenya to describe treatment choices made by rural households to treat a child's fever and the related costs to households. Using a cost-of-illness approach, we estimate the expected cost of a childhood fever to Kenyan households in 2002. We develop two scenarios to explore how expected costs to households would change if more children were treated at a health care facility with an effective antimalarial within 48 hours of fever onset.

**Results:**

30% of uncomplicated fevers were managed at home with modern medicines, 38% were taken to a health care facility (HCF), and 32% were managed at home without the use of modern medicines. Direct household cash expenditures were estimated at $0.44 per fever, while the total expected cost to households (cash and time) of an uncomplicated childhood fever is estimated to be $1.91. An estimated mean of 1.42 days of caretaker time devoted to each fever accounts for the majority of household costs of managing fevers. The aggregate cost to Kenyan households of managing uncomplicated childhood fevers was at least $96 million in 2002, equivalent to 1.00% of the Kenyan GDP.

Fewer than 8% of all fevers were treated with an antimalarial drug within 24 hours of fever onset, while 17.5% were treated within 48 hours at a HCF. To achieve an increase from 17.5% to 33% of fevers treated with an antimalarial drug within 48 hours at a HCF (Scenario 1), children already being taken to a HCF would need to be taken earlier. Under this scenario, direct cash expenditures would not change, and total household costs would fall slightly to $1.86 because caretakers also save time with prompt treatment if the child has malaria.

**Conclusion:**

The management of uncomplicated childhood fevers imposes substantial costs on Kenyan households. Achieving substantial improvements in the numbers of fevers treated within 48 hours at a HCF with an effective antimalarial drug (Scenario 1) will not impose additional costs on households. Achieving additional improvements in fevers treated promptly at a HCF (Scenario 2) will impose additional costs on some households roughly equal to average cash expenses for transportation to a HCF. Additional financing mechanisms that further reduce the costs of accessing care at a HCF and/or that make artemisinin-based combination therapies (ACTs) accessible for home management need to be developed and evaluated as a top priority.

## Background

Fever is the clinical hallmark of malaria disease. Diagnostic reliance on fever alone, however, results in high diagnostic sensitivity but poor specificity [1,2]. Nevertheless, as malaria is a common and frequently fatal disease among young children in sub-Saharan Africa, fever is regarded as a sufficient trigger for presumptive treatment of children with antimalarial drugs in most malaria endemic settings in Africa [3-5]. With more than 870 million fever events among African children in 2000 [6], the presumptive diagnosis of malaria in out-patient clinics across the continent occurs in between 30–40% of all consultations involving children [7].

The Roll Back Malaria (RBM) movement promotes prompt, effective treatment of fevers as a key strategy for achieving its optimistic mortality reduction goals [8]. RBM hopes to ensure that 80% of children receive effective antimalarial drugs within 24 hours of fever onset by the year 2010 [9]. International attention has, rightly, focused on the development [10], deployment [11,12], and financing of effective medicines [13-15], particularly artemisinin-based combination therapy (ACT). A separate and less considered issue is how communities will access these new medicines promptly and the costs to poor households that are located in rural areas far from health services.

Currently 32 countries in the World Health Organization (WHO) Africa Regional Office have changed, or are in the process of changing their first-line antimalarial drug policy to ACT [16]. Because very little pharmacovigilance data or oversight is available in Africa [17], however, the introduction of ACT has been cautious and confined largely to prescription-only distribution in the formal sector. This approach to the introduction of ACT could impose substantial additional costs on households if they need to switch from widely practiced home-based management [18] to seeking treatment at a HCF to access the new drugs.

In this paper, we assemble a series of data sets collected between 2001 and 2002 through clinical, epidemiological, and geographic studies on fever and malaria case-management in Kenya to describe the sequences of treatment choices made by rural households to treat a child's fever and the related costs to households. These data are used to estimate the expected cost of a childhood fever to Kenyan households. We then develop two scenarios to explore how household costs would change if a larger portion of children were treated within 48 hours of fever onset, given that artemether-lumefantrine (AL) is available in the formal health sector only.

## Methods

### Definitions and approach

Regardless of their underlying causes, fevers in children fall into three main categories: (1) uncomplicated fevers treated at home and/or through outpatient services at a health care facility (HCF) and clinically resolve; (2) complicated febrile events that progress to life-threatening conditions such as cerebral malaria and severe malaria anemia, requiring hospital care that if effective will result in cure; and (3) febrile events that lead rapidly to death because of rapid pathological progression before reaching tertiary care or failed treatment in tertiary care. The malaria parasite and its human hosts have adapted to ensure that uncomplicated fevers from malaria are extremely common whilst severe disease and death from malaria are comparatively rare [19]. Survey data used in the present analysis show that 99.6% of fevers among Kenyan children were either managed at home or as outpatients, while only 0.4% were admitted to hospital [20]. Thus, we focus our analysis here on the costs to households of uncomplicated fevers from all causes in children under five years.

We know that most fevers are not a direct consequence of malaria infection. The presence of any infection in children with fever presenting to clinics and retail outlets across malaria endemic areas of Africa may range from 2–64% depending on the intensity of malaria transmission [1,21-26]. However, the potential for serious and fatal outcomes developing rapidly from malaria if fevers in young, semi-immune children are not treated presumptively for malaria outweighs the costs of improved diagnosis prior to treatment [4,5].

Since households in Africa typically have to manage illnesses with fevers before knowing the primary cause, we focus this analysis on the costs to households of managing childhood fevers regardless of their cause. Using this household perspective, we also explore how such costs would change if these households followed international recommendations that all fevers should be treated promptly with an effective antimalarial in malaria endemic areas such as our study districts. In Kenya, AL began to be commonly available through government and mission HCFs in September 2006 at no additional cost to patients. Given this Kenyan policy, the primary costs to households from prompt treatment at a HCF with AL are changes in transportation costs and opportunity costs of caretaker time. Understanding these household costs is a prerequisite for designing appropriate mechanisms and incentives that facilitate the prompt delivery of febrile children to HCFs. Given our focus on costs to households, we emphasize here that were do not address related but separate topics, such as the costs to HCFs and the government of the adopted policy or the costs per illness event averted.

Throughout the paper, we use 'caretaker' to denote the person primarily responsible for child health care decision making (e.g., the mother). In practice, however, we realize that multiple caretakers may be involved in various decisions or providing care to children in the household.

We start with the assumption that a household acts as a joint decision making unit, adults are responsible for primary household decisions, and adults in the household care about their children's health. In this case, a household's willingness to pay (WTP) to avoid a fever in a child is conceptually a correct measure of the full cost of the fever to the household. Harrington and Portney [27] show that a household's WTP to avoid some unwanted health event comprises three components: (1) the costs actually borne by the household, including direct out-of-pocket expenses from the illness plus the monetary value of the time lost by household members (those ill and caretakers) while taking care of their ill child; (2) the value of averting expenditures made to avoid fevers in the first place; and (3) the monetary value of the disutility (pain and suffering) to the household and child from the illness.

Since the cost of illness to households can be regarded as a lower bound on the full cost to households as defined by WTP, and since reasonably high-quality data exist on the cost of illness, this paper follows a cost-of-illness approach to estimate the expected cost of a childhood fever to Kenyan households. And throughout this paper, we use the word average to imply the expected value/mean.

### Data sources

We used primary data from three surveys all conducted in late 2001 and early 2002 in four districts of Kenya: Greater Kisii, Kwale, Bondo, and Makueni [20,28-30]. The four districts are characteristic of the country's diverse malaria epidemiology and socio-economic situation. In all four districts, Kenyan national guidelines recommend presumptive treatment for malaria of all febrile children below 5 years of age. First, a survey of 9,272 randomly sampled homesteads between December 2001 and January 2002 collected detailed information on the management strategies and costs of 2,655 pediatric fever episodes experienced in the two weeks preceding the interview [20]. As noted in the definitions sections, we emphasize here that these are fevers from any cause, not fevers from malaria alone, and cause of the fever is unknown. For this analysis, we used data for the 1,565 uncomplicated fevers that had resolved by the day of interview, as these represent fevers with a complete history of treatment actions. We use these data to organize a model of home-based fever management as practiced by households and associated costs of care. Second, to estimate travel times to various service providers, we used data generated by non-Euclidian distance models derived from 668 households across the four districts [28]. And third, costs incurred by households at a HCF were derived from interviews with caretakers of 1,303 febrile children seen at 81 government clinics in the four districts during the health facility surveys undertaken at the same time as the community surveys [30]. Costs at a HCF include any costs of treatment, laboratory tests, and consultation fees incurred by the caretakers.

### Measuring the monetary value of caretaker time

Besides the actions taken by households to manage fevers, information on direct cash expenses paid by households during a fever and the monetary value or equivalent of caretaker time devoted to the child during a fever are needed for this analysis. Data on direct cash expenses paid by households for travel and treatment (both at home and at health care facilities) are directly available from the four district surveys [20,28-30]. To compare and add up with the same units, an estimate is needed of the monetary value of caretaker time devoted to their child during a fever.

The monetary value of caretaker time is a combination of two factors: how much time caretakers allocate to uncomplicated fevers; and the monetary equivalent of the value of caretaker's time. Regarding caretaker time, we could find no information in published or unpublished literature that documents the fraction of time caretakers reallocate from other activities to their children during an uncomplicated fever or other forms of mild illnesses. Given the focus here on uncomplicated fevers, we know that caretakers are able to continue their normal daily activities during much of the day when their child is febrile. How much is unknown, and this issue remains a good question for future research. As a starting point, or base case assumption, we assume that caretakers allocate an additional 0.25 days of their key productive time (2 hours per day assuming an 8 hour day) to their child during a fever. Due to our uncertainty around this parameter, we consider the implications of our analysis for higher and lower values in the discussion.

Besides the quantity of caretaker time per child fever, we also need an estimate of the monetary value of this time. In a non-wage economy such as the rural Kenya setting of this study, most income earning activities do not involve direct wage payments or cash incomes, so that information on the monetary value of caretaker time is difficult to obtain. Data are not directly available in the primary data discussed above for the four districts to estimate this value.

As a result, we developed an estimate of the monetary value of caretaker time based on a review of existing literature on poverty, adult daily income, and wages in Kenya. As a starting point, the World Bank reports for 2002 that national income was $11.1 billion, population was 31.3 million people, and per capita national income was $360 using the Atlas method for estimating income [31]. This low level of per capita income, roughly $1 per day, reflects well the country's rank (148th out of 177 countries) in the human development index of the United Nations Development Program [32].

Per capita income underestimates adult income because children are included in the calculation. However, per capita income overestimates average income because the distribution of income in Kenya is very skewed: 20% of the country's total population is estimated to earn 50% of national income [33]. We therefore estimated a daily income per adult based on national income for the bottom 80% of the country ($5.55 billion for 25.04 million people), which implies that per capita annual income for the poorest 80% of the population is $221. Using the average household size in the four study districts included in our analysis of 8.91 people (see data sources discussion above), average household income would be $1,975. Finally, using the average of 5 adults in households, average daily income per adult can be estimated at $1.08.

Using survey data from 2001, Dolan and Sutherland [34] report that the average daily wage for women horticultural workers was $1.31 (assuming an 8 hour shift). Given that there is substantial unemployment in Kenya and that a large percentage of women work at least part of their time in subsistence agriculture, this daily horticultural wage over-estimates the mean value of women's time whether in cash or non-cash production activities.

As another way of considering daily income, the Kenyan poverty line was defined by the government as $0.50 per day in 2002 [35], which overlaps the time period when the primary data discussed in the next section was collected. Precise information on the distribution of rural income in Kenya is not available for 2002 or other recent years. The World Bank does report that 53% of the rural population was below the national poverty line in 1997 [32]. Again using the average household size of 8.91 and 5 adults per household from the primary data referenced above, daily adult income would be $0.89 per day.

Based on the above discussion, we conclude that $1.00 per day provides a reasonable estimate of the average monetary value of caretaker time in the four districts of Kenya included in our study. Throughout the analysis, the 2002 annual average exchange rate of KES 78.30 per $1 US is used [36]. Because this analysis is developed for uncomplicated fevers in children under five years, we assume that the opportunity cost to the household of the child's time is zero. While not directly comparable, we note that $1 per day for adults was also used in a study of malaria costs in Ethiopia [37].

### Modeling average costs borne by households

Using the 1,565 fever events, we developed a simple decision tree to model the expected costs to households of a childhood fever. Decision trees are commonly used when estimating costs based on sequential decision making, including cost-effectiveness analysis related to malaria control [38,39]. While the decision tree approach used here is entirely consistent with such literature, our focus here is on the actions directly made by households related to fever case management for their children.

**Figure 1:**
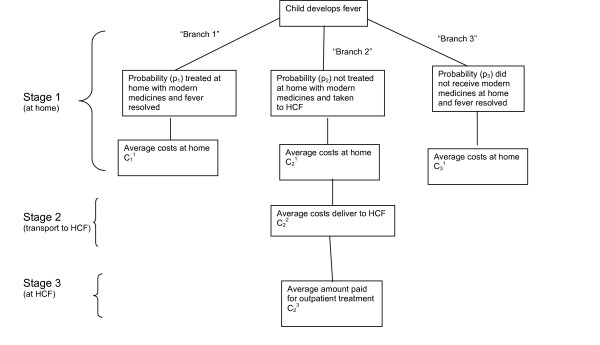
Household management of uncomplicated pediatric fevers in Kenya.

The model outlined in Figure 1 is organized into three management branches: treatment with modern medicines at home, such as antipyretics, antibiotics and antimalarials, where the fever resolves (Branch 1); delivery to a HCF without prior treatment with modern medicines at home (Branch 2); and management at home without modern medicines and the fever resolves (Branch 3). As shown in Figure 1, some percentage of fevers fall into each of the three branches, where *p*_1_, *p*_2_, and *p*_3 _are used to denote the proportion of fevers managed in each branch, with *p*_1 _+ *p*_2_*+ p*_3 _= 1.

Households incur costs of managing childhood fevers at three stages. Stage 1 covers all household actions and costs that occur at home, defined as the direct cash expenses for medicines and possibly other treatment costs and the value of caretaker time allocated to caring for the ill child. Stage 2 includes decisions and costs associated with delivering a child to a HCF and returning home, defined as cash expenses for transportation for the ill child and the caretaker and the value of caretaker time at this stage. Stage 3 covers all decisions and costs associated with obtaining outpatient care at a health care facility, defined as all fees paid by the caretaker for consultations, tests, and medicines and the opportunity costs of caretaker time while at a HCF and the additional time upon returning home until the fever resolves.

For notation, let C11
 MathType@MTEF@5@5@+=feaafiart1ev1aaatCvAUfKttLearuWrP9MDH5MBPbIqV92AaeXatLxBI9gBaebbnrfifHhDYfgasaacH8akY=wiFfYdH8Gipec8Eeeu0xXdbba9frFj0=OqFfea0dXdd9vqai=hGuQ8kuc9pgc9s8qqaq=dirpe0xb9q8qiLsFr0=vr0=vr0dc8meaabaqaciaacaGaaeqabaqabeGadaaakeaacqWGdbWqdaqhaaWcbaGaeGymaedabaGaeGymaedaaaaa@2FC8@, C21
 MathType@MTEF@5@5@+=feaafiart1ev1aaatCvAUfKttLearuWrP9MDH5MBPbIqV92AaeXatLxBI9gBaebbnrfifHhDYfgasaacH8akY=wiFfYdH8Gipec8Eeeu0xXdbba9frFj0=OqFfea0dXdd9vqai=hGuQ8kuc9pgc9s8qqaq=dirpe0xb9q8qiLsFr0=vr0=vr0dc8meaabaqaciaacaGaaeqabaqabeGadaaakeaacqWGdbWqdaqhaaWcbaGaeGOmaidabaGaeGymaedaaaaa@2FCA@, and C31
 MathType@MTEF@5@5@+=feaafiart1ev1aaatCvAUfKttLearuWrP9MDH5MBPbIqV92AaeXatLxBI9gBaebbnrfifHhDYfgasaacH8akY=wiFfYdH8Gipec8Eeeu0xXdbba9frFj0=OqFfea0dXdd9vqai=hGuQ8kuc9pgc9s8qqaq=dirpe0xb9q8qiLsFr0=vr0=vr0dc8meaabaqaciaacaGaaeqabaqabeGadaaakeaacqWGdbWqdaqhaaWcbaGaeG4mamdabaGaeGymaedaaaaa@2FCC@ represent expected costs in Stage 1 (actions at home) for each branch, and Stage 1 ends when either the child recovers or is taken to a HCF. Thus, the length of time associated with Stage 1 for each branch can vary. For children taken to a HCF (Branch 2), let C22
 MathType@MTEF@5@5@+=feaafiart1ev1aaatCvAUfKttLearuWrP9MDH5MBPbIqV92AaeXatLxBI9gBaebbnrfifHhDYfgasaacH8akY=wiFfYdH8Gipec8Eeeu0xXdbba9frFj0=OqFfea0dXdd9vqai=hGuQ8kuc9pgc9s8qqaq=dirpe0xb9q8qiLsFr0=vr0=vr0dc8meaabaqaciaacaGaaeqabaqabeGadaaakeaacqWGdbWqdaqhaaWcbaGaeGOmaidabaGaeGOmaidaaaaa@2FCC@ represent household costs for transportation and the value of caretaker time lost in the round trip to the facility, and let C23
 MathType@MTEF@5@5@+=feaafiart1ev1aaatCvAUfKttLearuWrP9MDH5MBPbIqV92AaeXatLxBI9gBaebbnrfifHhDYfgasaacH8akY=wiFfYdH8Gipec8Eeeu0xXdbba9frFj0=OqFfea0dXdd9vqai=hGuQ8kuc9pgc9s8qqaq=dirpe0xb9q8qiLsFr0=vr0=vr0dc8meaabaqaciaacaGaaeqabaqabeGadaaakeaacqWGdbWqdaqhaaWcbaGaeGOmaidabaGaeG4mamdaaaaa@2FCE@ represent costs at an HCF (Stage 3).

With the above notation, the expected value of costs to households of an uncomplicated fever in a child can be estimated as:

*E*(*C*) = *p*_1_C11
 MathType@MTEF@5@5@+=feaafiart1ev1aaatCvAUfKttLearuWrP9MDH5MBPbIqV92AaeXatLxBI9gBaebbnrfifHhDYfgasaacH8akY=wiFfYdH8Gipec8Eeeu0xXdbba9frFj0=OqFfea0dXdd9vqai=hGuQ8kuc9pgc9s8qqaq=dirpe0xb9q8qiLsFr0=vr0=vr0dc8meaabaqaciaacaGaaeqabaqabeGadaaakeaacqWGdbWqdaqhaaWcbaGaeGymaedabaGaeGymaedaaaaa@2FC8@ + *p*_3_C31
 MathType@MTEF@5@5@+=feaafiart1ev1aaatCvAUfKttLearuWrP9MDH5MBPbIqV92AaeXatLxBI9gBaebbnrfifHhDYfgasaacH8akY=wiFfYdH8Gipec8Eeeu0xXdbba9frFj0=OqFfea0dXdd9vqai=hGuQ8kuc9pgc9s8qqaq=dirpe0xb9q8qiLsFr0=vr0=vr0dc8meaabaqaciaacaGaaeqabaqabeGadaaakeaacqWGdbWqdaqhaaWcbaGaeG4mamdabaGaeGymaedaaaaa@2FCC@ + *p*_2 _(C21
 MathType@MTEF@5@5@+=feaafiart1ev1aaatCvAUfKttLearuWrP9MDH5MBPbIqV92AaeXatLxBI9gBaebbnrfifHhDYfgasaacH8akY=wiFfYdH8Gipec8Eeeu0xXdbba9frFj0=OqFfea0dXdd9vqai=hGuQ8kuc9pgc9s8qqaq=dirpe0xb9q8qiLsFr0=vr0=vr0dc8meaabaqaciaacaGaaeqabaqabeGadaaakeaacqWGdbWqdaqhaaWcbaGaeGOmaidabaGaeGymaedaaaaa@2FCA@ + C22
 MathType@MTEF@5@5@+=feaafiart1ev1aaatCvAUfKttLearuWrP9MDH5MBPbIqV92AaeXatLxBI9gBaebbnrfifHhDYfgasaacH8akY=wiFfYdH8Gipec8Eeeu0xXdbba9frFj0=OqFfea0dXdd9vqai=hGuQ8kuc9pgc9s8qqaq=dirpe0xb9q8qiLsFr0=vr0=vr0dc8meaabaqaciaacaGaaeqabaqabeGadaaakeaacqWGdbWqdaqhaaWcbaGaeGOmaidabaGaeGOmaidaaaaa@2FCC@ + C23
 MathType@MTEF@5@5@+=feaafiart1ev1aaatCvAUfKttLearuWrP9MDH5MBPbIqV92AaeXatLxBI9gBaebbnrfifHhDYfgasaacH8akY=wiFfYdH8Gipec8Eeeu0xXdbba9frFj0=OqFfea0dXdd9vqai=hGuQ8kuc9pgc9s8qqaq=dirpe0xb9q8qiLsFr0=vr0=vr0dc8meaabaqaciaacaGaaeqabaqabeGadaaakeaacqWGdbWqdaqhaaWcbaGaeGOmaidabaGaeG4mamdaaaaa@2FCE@)     (1)

Actions taken at home (Stage 1) can involve 'looping' within each branch or across branches [40,41]. For example, a caretaker might manage a fever initially through rest and comfort or herbal medicines (Branch 3). If the fever does not resolve, additional rest and or traditional medicines might be used (continuation of Branch 3), medicines purchased in retail outlets might then be given to the child (switch to Branch 1), or the child might be taken to an HFC (switch to Branch 2). Rather than developing a large series of management pathways to allow for all possibilities, the data available for each branch is consistent with the final actions taken by a household for each stage. So, for example, a child managed initially at home without medicines (Branch 3) who then received an antimalarial is considered to be in Branch 1 for this analysis.

We originally expected that some children treated at home with modern medicines (Branch 1) would have later been taken to a HCF, so that transportation and HCF costs for children managed in Branch 1 would need to be included in the decision tree. However, after a review of the data, none of the children managed in Branch 1 were later taken to a HCF. The decision tree outlined in Figure 1 can be easily adapted to analyze such situations when empirically relevant.

## Results

Table 1 presents initial parameter values and calculations used to estimate household costs of managing an uncomplicated fever event in children aged less than five years. Except for two parameters, caretaker time per fever and the monetary value of time, all parameters are means from the primary data sources. As discussed above, we assume initially in Table 1 that $1 (KES 78.30) is the daily value of caretaker time, and 0.25 days of caretaker time is allocated to child care for each sick day.

Table 1 is organized according to the three stages and three branches outlined in Figure 1. From the survey data generated in 2001–2002, 30% of uncomplicated fevers are managed at home with modern medicines (Branch 1), 38% are taken to a HCF (Branch 2), and 32% are managed at home without the use of modern medicines (Branch 3).

Regarding costs at home in Stage 1 from Table 1, fevers lasted on average for 4.8 days in Branch 1, 3.1 days in Branch 2, and 4.6 days in Branch 3. It is likely that the more serious fevers were managed in Branch 2 (taken to a HCF) since they were taken to a HCF on average at least 1.5–1.7 days earlier than it took for fevers to resolve in Branch 1 or Branch 3. Given the assumption that caretakers lost 0.25 days of productive time for each day of fever, we estimate in Stage 1 that caretakers allocated 1.20 days on average per fever in Branch 1, 0.78 days in Branch 2, and 1.15 days for Branch 3. For fevers managed in Branch 1, households spent on average KES 37.5 on average for modern medicines. Households did not have cash expenditures for managing fevers in Branch 3. In total, we estimate that average costs at home (Stage 1) are KES 131.46 for Branch 1, KES 60.68 for Branch 2, and KES 90.05 for Branch 3.

The same process is followed to estimate the average costs in Stage 2 for children taken to a HCF (Branch 2). Data on travel times to a HCF suggest a roundtrip travel time of 0.5 days, which also involves 0.5 days of caretaker time. Besides travel time, households on average spent KES 25 on direct travel expenses. In total, average costs in Stage 2 are KES 64.15.

In Stage 3 based on costs at a HCF, 87% of children were treated as outpatients with an antimalarial in 2002, and on average caretakers paid KES 35.70 in fees at a HCF per febrile child. Besides these direct cash expenses, the data show that a typical visit to a HCF lasts for 1.5 hours (0.18 days), and we assume that a fever resolves on average in two days after returning home (so 0.68 days of caretaker time at this stage). In total for Stage 3, the value of caretaker time and direct cash payments at a HCF are KES 89.32.

Across all three stages, household costs are estimated at KES 131.46 ($1.68) for fevers managed at home with modern medicines (Branch 1), KES 214.15 ($2.74) for fevers managed as outpatients at a HCF (Branch 2), and KES 90.05 ($1.15) for fevers managed at home without modern medicines (Branch 3). Table 1 also reports in KES and $US the main components of these costs (cash expenses, caretaker time, and the value of caretaker time). Using the branch probabilities reported in Table 1, the mean cost of an uncomplicated childhood fever to Kenyan households is estimated at KES 149.63 in 2002, or $1.91 at the average exchange rate for 2002.

Table 1 provides all parameters needed to estimate fever costs as outlined in Figure 1 and equation 1. As discussed above, the assumption that 0.25 days of caretaker time is devoted to each fever is the only figure that is not based on primary data sources or at least fairly solid secondary sources (the $1 per day figure for the value of caretaker time). Given the uncertainty around the amount of caretaker time allocated per fever, Table 2 summarizes the results of estimating the model with a higher and lower figure for this parameter (0.5 and 0.125). While 0.5 days per fever as a mean across all uncomplicated fevers is probably too high, we suspect that 0.125 days is too low. For each of these assumptions on caretaker time provided in Table 2 under "Current Practice", direct cash expenditures remain $0.44. Caretaker time ranges from 1.47 days for the base case to 0.86 and 2.68 days for the lower and higher range. Combining direct cash expenses and the value of time, estimated fever costs are $1.30 for the lower estimate, $1.91 for the base case, and $3.12 for the higher estimate.

As shown in Table 2, the value of caretaker time accounts for 77% of estimated costs for the base case (0.25 days of caretaker time per fever day, and 1.47/1.91 = 0.77 or 77%), and 66% of estimated costs for the lower range (0.125 days caretaker time per fever day). Total cash expenses of $0.44 on medicines at home, transportation to a HCF, and fees at a HCF (consultation, laboratory, and drugs) are equal to 23% of costs in the base case (0.44/1.91 = 0.23 or 23%) and 34% of costs for the lower range.

For perspective on the annual implications for households of childhood fevers, the most recent 2003 Kenya Demographic and Health Survey reported that 40.6% of children under five had a fever in the two weeks prior to the interview [42]. Multiplying this two-week incidence by 26, children in Kenya experience on average 10.5 fevers annually. Thus, using the base case results of $1.91 per fever from Table 1 and Table 2, the annual average cost to households of fevers in one child is estimated to include $4.62 in direct cash expenses and 15.44 days of caretaker time, for a total of $20.06 annually. For the lower range of 0.125 days of caretaker time per fever day, cash expenses remain at $4.62, caretaker time falls to 9.03 days, and total annual average fever costs per child are $13.65.

### ACT policy change scenarios

In Kenya one specific ACT, Artemether-lumefantrine (AL), began to be available through government and mission HCFs between July and September 2006 at no additional costs to patients. Existing data suggest that Kenya is far from achieving the "80% within 24 hours" target set by RBM: our data show that fewer than 8% of fevers were treated with an antimalarial within 24 hours of onset in 2001/2002. We emphasize here that this RBM target is defined for all fevers in children under 5, not just fevers from malaria. In practice this definition of within 24 hours is hard to define reliably through field investigation, and a more pragmatic target would be within 48 hours or same-day/next-day access to effective drugs. Using this extended period as a yard-stick of theoretical programme success, only 17.5% of fevers were treated with an antimalarial within 48 hours at a HCF [20].

Here we investigate the implications for Kenyan households of achieving modest progress towards the RBM targets, from the current 17.5% to 33% and 50% of all fevers receiving an antimalarial within 48 hours at a HCF, with AL as the first-line recommended therapy. We provide two scenarios for achieving this progress. For both scenarios we assume that AL is introduced into the formal health sector only, which is the Kenyan policy.

#### Scenario 1 – earlier delivery to a HCF for children already in Branch 2

As reported in Branch 2 of Table 1, current practice delivers 38% of fevers to a HCF, and fevers in this branch are estimated to require 1.96 days of caretaker time. For these children, only 53% were taken to a HCF within 48 hours of fever onset, and only 87% of those taken actually received an antimalarial drug (see Table 1, Stage 3 information). In total, under current practice, 17.5% of all children with fever are treated with an antimalarial at a HCF (0.38*0.53*0.87 = 0.175)

To reach a target of 33% of all fevers treated with AL within 48 hours when AL is only available at a HCF, which is almost a 100% improvement on current practice, all the children already being taken to a HCF (Branch 2) in Table 1 would need to depart home within 1.75 days of fever onset, with an additional 0.25 days of transport time (2 hours), so that the child arrives at a HCF within 2 days. The first implication of this scenario (Scenario 1) is that caretaker time in Stage 1 for Branch 2 would fall from 0.78 days in Table 1 to 0.44 days.

For this scenario, cash expenses do not change because the same people travel to a HCF. Costs at a HCF are also not expected to change with the availability of AL because the government has proposed to provide it free to all pediatric patients.

While children in Branch 2 are delivered to a HCF on average 1.35 days earlier for Scenario 1 than current practice as reported in Table 2, with a saving of 0.34 days of caretaker time in Stage 1, earlier delivery to a HCF would only be expected to reduce the overall illness time if the child has malaria. For children delivered earlier in Branch 2 who do not have malaria, receiving AL should have no impact on the length of their fever. For these children, we would continue to expect on average that caretakers devote 1.96 days of time to managing their child's fever (as in Table 1 for Branch 2). In this case, earlier delivery to a HCF that saves 0.34 days of caretaker time in Stage 1 would simply imply a longer fever resolution time (and 0.34 days of caretaker time) in Stage 3 at home after a HCF visit.

Given the initial estimate of 0.68 days of caretaker time at a HCF and at home after the visit for Branch 2 in Table 1, we would now expect 0.68 + 0.34 = 1.02 days would be the appropriate amount of caretaker time in Stage 3 for children delivered to a HCF earlier without malaria. For children taken earlier to a HCF earlier who have malaria, earlier treatment should imply earlier resolution of the fever. For these children, the estimate of 0.68 days of caretaker time at a HCF and at home following remains the correct figure. Assuming that only 40% of children with fever in Branch 2 actually have malaria, on average 0.40*0.68 + 0.60*1.02 = 0.88 days of caretaker time is the appropriate figure to include for caretaker time in Stage 3 for Scenario 1.

As reported in Table 2, reducing the time before delivery to a HCF from 3.1 days to 1.75 days at home (Stage 1), but not changing treatment seeking behavior of specific households, reduces caretaker time on average per fever across all three branches from 1.47 days for the base case to 1.42 days for Scenario 1 (and from 1.96 days to 1.82 days for fevers in Branch 2). Given constant cash costs, the average cost of a fever in Scenario 1 falls slightly to $1.86.

This scenario assumes that the availability of more effective medicines at HCFs would lead caretakers to change their treatment seeking behavior to access treatment within the 48-hour target. Whether this behavioral change is possible depends on if community awareness campaigns about AL are effective and word-of-mouth community recognition of drug effectiveness affects behavior.

As shown in Table 2, the same qualitative results comparing the base case with Scenario 1 hold whether 0.25, 0.125, or 0.5 days of caretaker time per fever day is used for the analysis. Cash expenses do not change and some caretakers save time. As a result, the overall average cost per fever to households fall with earlier delivery to a HCF.

#### Scenario 2 – changing treatment seeking behavior

If we raised the numbers of children effectively managed with AL within 48 hours from 33% of all fevers, as in Scenario 1, to 50% (Scenario 2), treatment seeking behavior would also need to change so that a larger percentage of all fevers were taken to a HCF and prescribed AL. If the percentage of fevers treated at home fell from 30% to 10.5%, for example, and instead these 19.5% of fevers were delivered to a HCF within 48 hours, 57.5% of all fevers would be managed in Branch 2 and 50% of all fevers would be treated with AL at a HCF (assuming as in Table 1 that 87% of fevers at a HCF are prescribed AL).

The first implication of Scenario 2 is that cash expenses would increase by $0.30 for the 19.5% of all fevers switched from Branch 1 to Branch 2. In Table 1, cash expenses are $0.48 for fevers managed in Branch 1 because households purchased medicines used to manage these fevers, while cash expenses are $0.78 for fevers managed in Branch 2 because of transportation costs and fees at a HCF. The difference between the two branches of $0.30 is essentially equal to cash expenses for transportation in Branch 2.

In Scenario 2, the majority of children (38% of all fevers out of the total 57.5% of all fevers in branch 2) are the same children analyzed as part of Scenario 1 and, therefore, the numbers for their analysis do not change as part of Scenario 2. Fevers for these children are estimated to require 1.82 days of caretaker time on average (0.44 days in Stage 1, 0.50 days in Stage 2, and 0.88 days in Stage 3).

The 19.5% out of 57.5% of all fevers now in Branch 2 for Scenario 2 are children who were managed in Branch 1 (treated at home). As reported in Table 2, these fevers resolved on average in 4.8 days, with an estimate of 1.20 days of caretaker time. Thus, fevers managed in Branch 1 resolved more quickly and required less caretaker time (1.2 days of caretaker time) than fevers managed in Branch 2 (1.82 days on average in Scenario 1). Delivery to a HCF should not increase the overall time for these children's fevers to resolve (fevers switched from Branch 1 to Branch 2). Assuming that 0.44 days of caretaker time are used in Stage 1 and 0.50 days for Stage 2, we would estimate that 1.20 – 0.44 – 0.50 = 0.26 days is the maximum average amount of caretaker time used in Stage 3 for the fevers switched from Branch 1 to Branch 2. The 0.26 days figure is a maximum because some of the fevers treated earlier would be malaria, as discussed in Scenario 1, so that their fever resolution time could fall with earlier AL treatment. Thus, for all of the fevers managed in Branch 2 for Scenario 2, on average (0.195/0.575)0.26 + (0.38/0.575)0.88 = 0.67 days of caretaker time would be used as the overall average amount of caretaker time in Stage 3 of Branch 2 for Scenario 2.

As shown in Table 2, the average cost of a fever across all three branches is $1.92 for Scenario 2. Changing treatment seeking behavior as considered in this scenario would involve a $0.06 increase in direct cash expenses per fever, and a slight decrease in days of caretaker time (0.05 days for the base case and the lower range). As discussed above, however, all of the average cash cost increase comes from a cash cost increase of $0.30 for the 19.5% of fevers switched from Branch 1 to Branch 2.

Scenario 2 assumes that the majority of caretakers who previously managed their children at home with modern medicines (branch 1) would now instead bring their children to a HCF. Again, whether this behavioral change is possible depends on if community awareness campaigns are effective and word-of-mouth community recognition of drug effectiveness affect behavior.

## Discussion

We have estimated that the average cost of managing a single uncomplicated pediatric fever to Kenyan households in 2002 was $1.91 based on a model of multiple treatment pathways at different stages of a febrile event. This estimate is based on primary data across four areas of Kenya and the assumption that each fever day in a child requires 0.25 days on average of caretaker time. If only 0.125 days of caretaker time are required on average per fever day, average costs remain $1.30. Annually, the cost to households of fevers in one child for both the 0.25 and 0.125 assumption are estimated at $20.06 and $13.65, which includes cash expenses of $4.62 for both and 15.44 and 9.03 days caretaker time respectively.

As would be expected, there are a range of costs around this average. Some fevers are managed at home without additional medicines, last for a short time, involve little or no additional adult caretaker time, and as a result entail almost zero cost. On the other hand, if a child remains at home for several days and is then taken to a facility, the cost of such a fever could be in excess of $4.00, with the opportunity cost of caretaker time still accounting for the vast majority of the total.

The most recent 2003 Kenya Demographic and Health Survey of 10,000 households in 400 clusters located across Kenya's eight provinces reported that 40.6% of children under five had a fever in the two weeks prior to the interview, with a low of 23.2% in North Eastern Province and a high of 59.8% in Western Province [42]. Projecting Kenya's provincial population data from the last national census in 1999 through to 2005, using the provincial fever prevalence estimates from the 2003 KDHS, and assuming a lower annual incidence as 26 times the two week period prevalence estimate [6], we estimate that there were 55.53 million childhood fevers in 2005 and conservatively estimate that 50 million of these would be uncomplicated. Assuming that the number of fevers in 2002 was essentially the same as 2005, and using the $1.92 cost per fever from Table 1, the aggregate cost to Kenyan households of managing uncomplicated childhood fevers was $96 million in 2002, representing a direct cost to the Kenyan economy equivalent to 1.00% of annual Kenyan gross 2002 national income.

Our data and modeled estimates of cost presented in Table 1 and Table 2 under Current Practice represent a situation that prevailed before the introduction of ACTs in Kenya. During the era of failing drugs, only 17.5% of fevers were managed at a HCF within 48 hours, presenting a double challenge for effective malaria case-management in febrile children: ineffective drugs and poor access. The new drug policy was implemented in 2006 and is limited to the formal health sector.

We have examined two approaches for increasing effective coverage of new medicines when these are limited to the formal health sector. The first approach, Scenario 1, is to reduce the time between fever onset and delivery to a HCF to 48 hours for the 38% of fevers already being taken to a HCF. As shown in Table 2, achieving this change in behavior would increase the percentage of children receiving an effective antimalarial from 17.5% to 33%, cash expenses would not change, and the average cost of a fever would fall slightly to $1.86, with all of the cost savings from reducing the value of caretaker time.

The second, complementary approach (Scenario 2) would be to seek further behavioral change where more caretakers manage the febrile child at a HCF rather than manage at home with retail sector drugs. While the average cost across all fevers in Scenario 2 remains essentially the same as estimated as current practice, Scenario 2 does involve a shift from cash expenses at home for medicines to cash expenses for transportation and fees at a HCF, with a net increase of $0.30 per fever for the 19.5% of fevers switched from home management to care at a HCF.

With the RBM goal of prompt treatment of 80% of all childhood fevers at a HCF by 2010, the analysis presented here for Scenario 1 shows that Kenyan can achieve substantial progress towards this target by focusing on earlier delivery of children already being taken to a HCF. In other words, the first 41% (33% out of 80%) of the RBM target can be achieved without imposing additional costs on households. To achieve further progress, Scenario 2 shows that 63% of the RBM target (50% of all fevers out of 80%) could be achieved in Kenya with an estimated $0.30 increase in cash expenses for 19.5% of all fevers.

While making new medicines more accessible over-the-counter (OTC) in the retail sector could save caretakers travel time and expenses for fever management [26,43], unsubsidized retail prices of KES 300 or more for a child ACT course are prohibitive for poorer households. If the situation described for 2001/2002 prevails in 2009, when AL is anticipated to be deregulated for OTC use in Kenya, but the cost of drugs is not centrally subsidized, the average costs to households would increase substantially if retails purchases of ACT increased substantially. We doubt, however, that caretakers formerly managing fevers at home with inexpensive retail medicines would switch and actually purchase new medicines at unsubsidized prices. In the absence of highly subsidized prices at the retail level, increasing this percentage through OTC access is highly unlikely.

## Conclusion

These analyses highlight several implications of changing malaria treatment policy in Kenya. First, limiting new drugs to a single sector and aiming to increase prompt access will not increase opportunity costs to impoverished households who are already taking their children to a HCF (Scenario 1). On average, prompt access will shorten time managing a fever at home for fevers associated with malaria. Second, the only option to increase prompt access when new drugs are limited to a single sector is to encourage people to travel earlier to service providers, representing investment in major behavioral change initiatives at community levels. Such initiatives need to differentiate messages to target, for example, households who tend to take their children to a HCF but later than recommended (e.g., after two days), households who tend to manage fevers at home but could access a HCF if required for little or no cash expense, and households who manage fevers at home but can only access a HCF with significant cash expenses. And third, unless new drugs are heavily subsidized in the retail sector, achieving more than 50% of all fevers treated promptly at a HCF (63% of the RBM target of 80%) would increase substantially the average costs to households of managing pediatric fevers in Kenya. For Kenya to achieve substantial increases beyond Scenario 2, the international community and Kenyan government must find financing options that make the cost of accessing new ACT drugs more affordable to poor rural households [14].

## Competing interests

The author(s) declare that they have no competing interests.

## Authors' contributions

BAL contributed to the conception and design of this study, analysis and interpretation of results, and drafting/completion of the paper.

AAA contributed to the conception and design of this study, analysis and interpretation of results, and drafting/completion of the paper.

AMN contributed to the conception and design of this study, analysis and interpretation of results, and drafting/completion of the paper.

DZ contributed to the development of basic data used in this study, analysis and interpretation of results, and drafting/completion of the paper.

RWS provided overall guidance, advice, and input into the analysis, interpretation, and writing of the paper.

All authors have read and approved the final manuscript.

## Pre-publication history

The pre-publication history for this paper can be accessed here:



## Figures and Tables

**Table 1:** The average cost of a childhood fever in Kenya

	Units	Branch 1	Branch 2	Branch 3
Branch Probabilities	probability ^1	0.30	0.38	0.32

**Stage 1. Costs at Home**				
Child Days Sick This Stage	Days ^1	4.80	3.10	4.60
Caretaker Time This Stage	Days ^2	1.20	0.78	1.15
Value of Caretaker Time	KES/Day ^2	78.30	78.30	78.30
Total Value of Caretaker Time This Stage	KES	93.96	60.68	90.05
Cost Of Medicines and/or Other Treatments This Stage	KES ^1	37.50	0.00	0.00
Total Cost Stage 1	KES	131.46	60.68	90.05
				
**Stage 2. Cost of Travel to a HCF (Round Trip)**				
Caretaker Travel Time (Round Trip)	Days ^3		0.50	
Value of Caretaker Time	KES/Day ^2		78.30	
Total Value of Caretaker Time Stage	KES		39.15	
Cash Travel Expenses (Caregiver + Child)	KES ^3		25.00	
Total Cost Stage 2	KES		64.15	
				
**Stage 3. Costs at a HCF**				
% Treated with AM	probability ^4		0.87	
Average cost if treated with AM	KES^4		35.70	
% Not treated with AM	probability^4		0.13	
Average cost if not treated with AM	KES^4		38.60	
Average Cost at HCF	KES		36.08	
Caretaker Time at a HCF and at home until fever resolves	Days ^2		0.68	
Value of Caretaker Time	KES/Day ^2		78.30	
Total Costs Stage 3	KES		89.32	
				
**Mean cost of fever managed in each branch**	KES	131.46	214.15	90.05
	$	1.68	2.74	1.15
of which				
direct cash expenditures	KES	37.50	61.08	0.00
	$	0.48	0.78	0.00
days of caretaker time	Days	1.20	1.96	1.15
opportunity cost of caretaker time	KES	93.96	153.08	90.05
	$	1.20	1.96	1.15

**Mean cost of fever**	KES	149.63		
	$	1.91		

Notes to Table:

^1 Amin et al. [20]

^2 Assumption discussed in text.

^3 Noor et al. [28]

^4 Zurovac et al. [30]

**Table 2:** Fever costs by key components with alternative caretaker time assumptions

		Base Case From Table 1.	Lower Range	Higher Range
		0.25 days caretaker time per fever day	0.125 days caretaker time per fever day	0.5 days caretaker time per fever day

**Current Practice:**	Units			
direct cash expenditures	$	0.44	0.44	0.44
days of caretaker time	Days	1.47	0.86	2.68
value of caretaker time	$	1.47	0.86	2.68
total (cash and time)	$	1.91	1.30	3.12
				
**Scenario 1**: 33% of fevers treated within 48 hours at a HCF with ACT				
direct cash expenditures	$	0.44	0.44	0.44
days of caretaker time	Days	1.42	0.84	2.58
value of caretaker time	$	1.42	0.84	2.58
total (cash and time)	$	1.86	1.28	3.02
				
**Scenario 2**: 50% of fevers treated within 48 hours at a HCF with ACT				
direct cash expenditures	$	0.50	0.50	0.50
days of caretaker time	Days	1.42	0.84	2.58
value of caretaker time	$	1.42	0.84	2.58
total (cash and time)	$	1.92	1.34	3.08
